# STATISTICAL MODELS FOR ESTIMATING RESILIENCE

**DOI:** 10.1093/geroni/igad104.1826

**Published:** 2023-12-21

**Authors:** Carl Pieper, Cathleen Colon-Emeric, Richard Sloane, Sarah Peskoe, Heather Whitson

**Affiliations:** Duke University School of Medicine, Durham, North Carolina, United States; Duke University, Durham, North Carolina, United States; Duke University Medical Center, Durham, North Carolina, United States; Duke University School of Medicine, Durham, North Carolina, United States; Duke University, Durham, North Carolina, United States

## Abstract

One important component of the Resilience paradigm is the degree of initial change and rapidity of recovery after a stressor. Resilience is determined by both the degree of response, if any, to an insult and, subsequently, the process of return to the initial state. Modeling the recovery process requires use of longitudinal analysis which can incorporate the underlying non-linear trajectory of change, and the multiple systems impacted by the stressor. We will show the models we have employed for one common stressor, surgery. First, we will define and show findings on the Expected Predicted Differential (ERD), the difference between predicted and observed recovery. Second, we will show multivariate trajectories of recovery to demonstrate methods to (1) define typologies of recovery, using PCA, and (2) typologies of individuals in type of recovery, using latent class analysis of trajectories. Second, given the state of the emerging art in resilience research, we will provide a statistician’s view on enhancing the resilience research through design (increasing number and timing of measurements, the number of subjects, and expanding use of the EHR), data structures (incorporating measurements from a greater number of diverse sources, using timing of measures to allow for assessment of dynamic mediation and moderation effects in defining the process), and analysis - extending the ERD to modeling non-linear recovery, incorporating causal analysis, including dynamic mediating and moderating effects into the analytic structure, use Area Under the Curve as a metric for recovery, latent class analysis, and use of multivariate trajectory analysis.

